# Supplementary figures and images for: 16S rRNA Gene Sequencing Revealed Changes in Gut Microbiota Composition during Pregnancy and Lactation in Mice Model
Source: Vet Sci. 2022 Apr 1;9(4):169. doi: 10.3390/vetsci9040169 (PMC9024687; doi:10.3390/vetsci9040169)

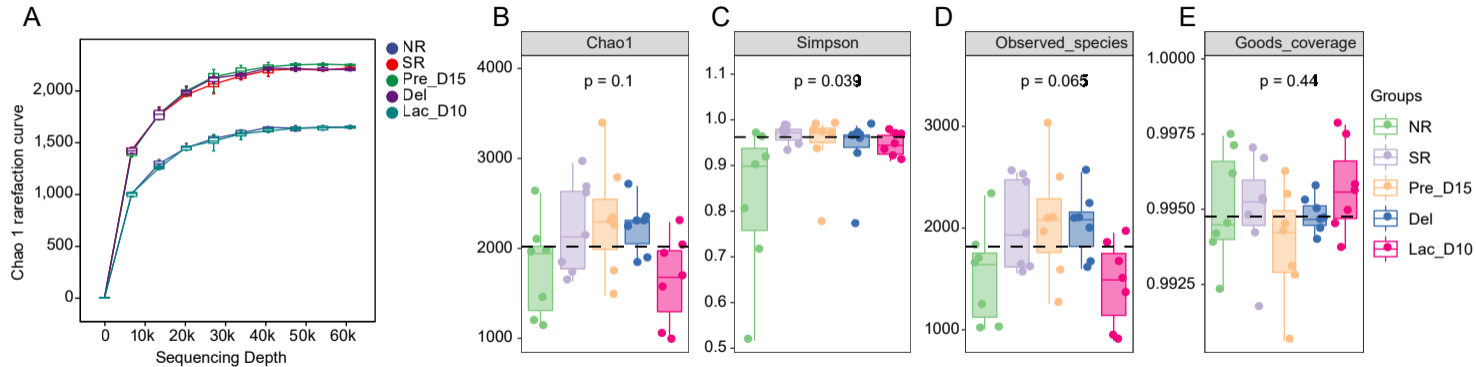

Supplement: Supplementary file 1 [file vetsci-09-00169-s001.zip › vetsci-1626994-supplementary/Supplemental files/Supplemental Figure S1.pdf]

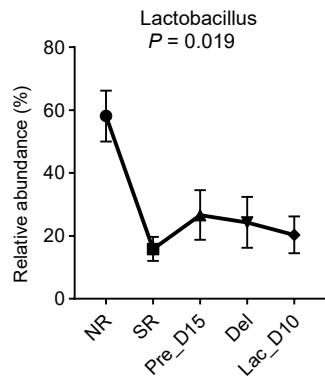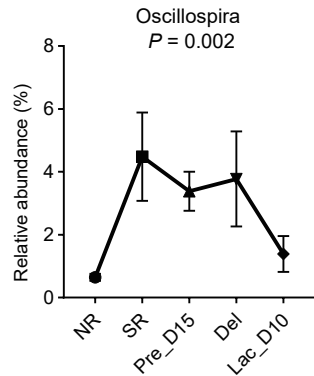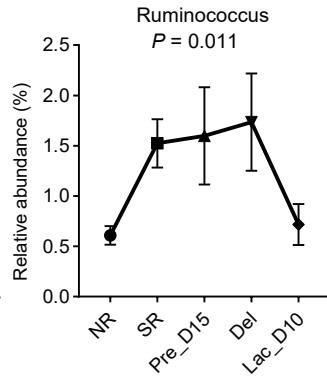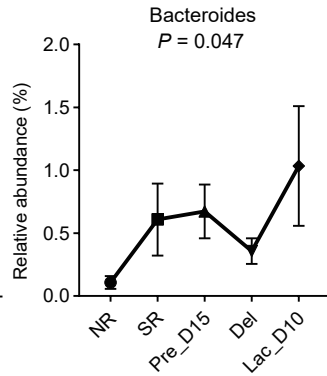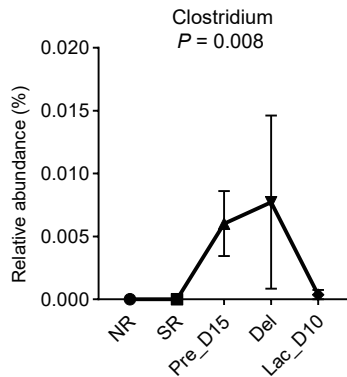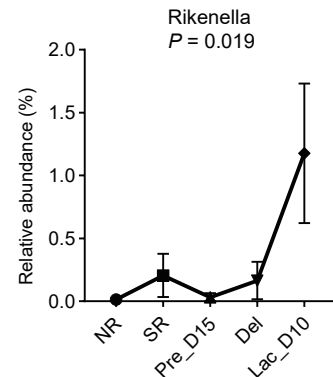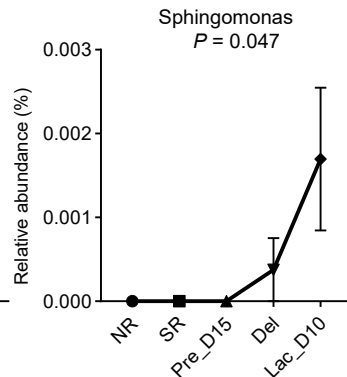

Supplement: Supplementary file 1 [file vetsci-09-00169-s001.zip › vetsci-1626994-supplementary/Supplemental files/Supplemental Figure S2.pdf]
